# Melatonin Alleviates Behavioral and Neurodevelopmental Abnormalities in Offspring Caused by Prenatal Stress

**DOI:** 10.1111/cns.70347

**Published:** 2025-03-25

**Authors:** Dong Wu, Jingyi Du, Tiantian Zhao, Naigang Li, Xinghui Qiao, Fan Peng, Dongshuang Wang, Jiaming Shi, Shu Zhang, Can Diao, Liyan Wang, Wenjuan Zhou, Aijun Hao

**Affiliations:** ^1^ Key Laboratory for Experimental Teratology of Ministry of Education, Shandong Key Laboratory of Mental Disorders and Intelligent Control, Department of Anatomy and Histoembryology, School of Basic Medical Sciences Cheeloo College of Medicine, Shandong University Jinan China; ^2^ School of Basic Medical Sciences Cheeloo College of Medicine, Shandong University Jinan Shandong China

**Keywords:** CXCL10, melatonin, microglia, neurogenesis, PI3K/AKT/NF‐κB, prenatal stress, synapse

## Abstract

**Background:**

Prenatal stress (PNS) is a significant risk factor impacting the lifelong health of offspring, and it has been widely recognized as being closely linked to the increased prevalence of neurodevelopmental disorders and psychiatric illnesses. However, effective pharmacological interventions to mitigate its detrimental effects remain limited. Melatonin (Mel), an endogenous hormone, has demonstrated considerable potential in treating neurological diseases due to its anti‐inflammatory, antioxidant, and neuroprotective properties, as well as its favorable safety profile and broad clinical applicability.

**Objective:**

This study aims to investigate the protective effects and mechanisms of melatonin on neurodevelopmental and behavioral abnormalities in offspring induced by prenatal stress.

**Methods:**

Using a prenatal stress mouse model, we evaluated the effects of melatonin on emotional and cognitive deficits in offspring. Neurogenesis and synaptic development were assessed, and RNA sequencing was performed to analyze microglial gene enrichment and immune‐related pathways. Both in vivo and in vitro experiments were conducted to validate the findings, focusing on the PI3K/AKT/NF‐κB signaling pathway in microglia.

**Results:**

Melatonin administration alleviated emotional and cognitive deficits in offspring mice exposed to prenatal stress, addressing abnormalities in neurogenesis and synaptic development. Additionally, RNA sequencing revealed that melatonin suppresses microglial gene enrichment and the upregulation of immune‐related pathways. Both in vivo and in vitro validation indicated that melatonin modulates the PI3K/AKT/NF‐κB signaling pathway in microglia, reducing the elevated expression of CXCL10 in the dentate gyrus, thereby restoring normal neuro‐supportive functions and optimizing the neurodevelopmental environment.

**Conclusion:**

These findings suggest that melatonin significantly improves neurodevelopmental disorders and behavioral abnormalities caused by prenatal stress by inhibiting pathological microglial activation and promoting hippocampal neurogenesis and synaptic plasticity. This provides new insights into melatonin's potential as a neuroprotective agent for treating prenatal stress‐related disorders.

## Graphical abstract

Prenatal stress increases the risk of neurodevelopmental disorders and psychiatric illnesses in offspring. This study demonstrates that melatonin alleviates emotional and cognitive deficits in offspring exposed to prenatal stress. By modulating the PI3K/AKT/NF‐κB signaling pathway in microglia and reducing the elevated expression of CXCL10 in the dentate gyrus, melatonin promotes neurogenesis and synaptic plasticity.

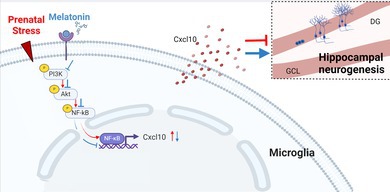

## 1 Introduction

Mothers undergo significant physiological and psychological changes during pregnancy, making them more susceptible to various sources of stress, such as life event stress, partner relationship tensions, and external conflicts or natural disasters [1, 2, 3]. Research over the past two decades has shown a strong correlation between prenatal stress and an increased risk of emotional, behavioral, and cognitive problems in both children and adults, including anxiety, depression, attention deficit hyperactivity disorder (ADHD), conduct disorder, and autism spectrum disorder (ASD) [4, 5, 6, 7]. Animal studies have indicated that prenatal stress significantly impacts neurodevelopment, altering the brain structure and function of offspring [8, 9, 10, 11].

Prenatal stress may interfere with neurodevelopment through mechanisms such as the hypothalamic–pituitary–adrenal (HPA) axis, oxidative stress, and inflammation [12, 13, 14]. Preclinical investigations have revealed that the hippocampus, characterized by a high density of glucocorticoid receptors, is particularly sensitive to early stress [15, 16]. Furthermore, research shows that prenatal stress‐induced neuronal and synaptic changes are highly region‐specific, particularly in areas involved in cognitive and emotional functions, such as the prefrontal cortex and limbic system (e.g., hippocampal CA1, CA3, and dentate gyrus), which exhibit particularly marked responses to stress exposure [17, 18, 19]. Numerous studies have shown that offspring of rats and nonhuman primates repeatedly exposed to prenatal stress exhibit significant changes in hippocampal structure, including reduced neurogenesis and altered synaptic plasticity, both in the neonatal and adult stages [20, 21, 22].

Microglia, the macrophages of the central nervous system (CNS), originate from the yolk sac and migrate to the brain parenchyma on embryonic Day 9.5, playing a crucial role in CNS development and homeostasis [23, 24]. They are key regulators of neurogenesis and synaptic plasticity, promoting the proliferation and differentiation of neural stem cells by releasing cytokines and growth factors, and adjusting the structure and connectivity of neural networks by phagocytosing excess synapses [23, 25]. Microglia are highly sensitive to external stressors (e.g., infection, glucocorticoids), capable of releasing pro‐inflammatory signals and interacting with neighboring cells to regulate neuroinflammation [26]. Studies have shown that early life stress can alter the distribution and function of microglia [27, 28], which may be a key factor in the development of neurodevelopmental disorders, making microglia a therapeutic target for CNS development and related diseases.

Melatonin, an endogenous free radical scavenger secreted by the pineal gland, has various physiological functions, including antioxidant, skeletal protection, reproductive regulation, and cardiovascular and immune functions [29, 30, 31, 32, 33]. Melatonin also demonstrates therapeutic potential in neuroprotection by modulating oxidative stress and neuroinflammatory responses, scavenging free radicals, reducing oxidative stress, and inhibiting microglial activation, thereby alleviating neurological damage caused by inflammation [34, 35]. Clinical studies have shown that melatonin levels increase in healthy women during pregnancy [36, 37]. Both the embryo and the newborn depend on maternal melatonin levels, which cross the placental barrier, with fetal circadian rhythms following those of the mother [38, 39]. Maternal melatonin levels are critical for normal fetal development [40, 41]. Prenatal stress disrupts maternal circadian rhythms, resulting in lower nighttime plasma melatonin concentrations in depressed pregnant women compared to healthy pregnant women [42, 43]. Therefore, it is of great significance to pay attention to the mental state and circadian rhythm of pregnant women and maintain the stability of melatonin levels in pregnant women for the healthy development of the fetus.

At present, given the lack of effective medications to regulate maternal prenatal stress and the high recognition of melatonin as an endogenous endocrine hormone for its safety and efficacy, it is worth paying attention to whether it can be used to treat prenatal stress. Given the potential role of melatonin in addressing prenatal stress‐induced neurodevelopmental abnormalities, we established a mouse prenatal stress model to investigate whether melatonin can ameliorate these abnormalities and explored the role of microglia in thisprocess. This research aims to provide insights into the therapeutic potential of melatonin in addressing prenatal stress and its impact on neurodevelopment, thereby contributing to the development of effective interventions for maternal and fetal health.

At present, given the lack of effective medications to regulate maternal prenatal stress and the high recognition of melatonin as an endogenous endocrine hormone for its safety and efficacy, it is worth paying attention to whether it can be used to treat prenatal stress. Given the potential role of melatonin in addressing prenatal stress‐induced neurodevelopmental abnormalities, we established a mouse prenatal stress model to investigate whether melatonin can ameliorate these abnormalities and explored the role of microglia in this process. This research aims to provide insights into the therapeutic potential of melatonin in addressing prenatal stress and its impact on neurodevelopment, thereby contributing to the development of effective interventions for maternal and fetal health.

## 2 Material and Methods

### 2.1 Animals

The experiment was conducted using C57BL/6 mice, purchased from Beijing Vital River Laboratory Animal Technology Co. Ltd. The mice were housed in an animal facility maintained at a constant temperature of 22°C ± 2°C and humidity (45%–55%), with a 12‐h light/dark cycle (06:00–18:00), and allowed ad libitum access to food and water. All animal care and experiments were conducted in accordance with the Guide for the Care and Use of Laboratory Animals by the National Institutes of Health and were approved by the Ethics Committee on Animal Experiments of the Medical School of Shandong University. The animals were monitored by trained veterinary personnel. Every effort was made to minimize the number of animals used and to reduce pain or discomfort during the experiments.

### 2.2 Prenatal Stress Model

Two female mice were paired with one male per cage for breeding until the appearance of a vaginal plug, marking embryonic Day 0.5 (E0.5). On E0.5, pregnant mice were randomly assigned to the Control, PNS, and PNS + Mel groups and housed individually in plastic cages.

To induce prenatal stress, pregnant mice were placed in a plexiglass restraint apparatus and under bright lights (2400LUX) for 45 min, three times a day (7:30–8:15 AM, 11:30–12:15 PM, and 2:30–3:15 PM), starting on E9.5 and continuing for 10 days. This protocol was based on a previously described method with minor adaptations [44, 45, 46]. The day of offspring birth was designated as postnatal Day 1 (P1). Offspring were weaned from their mothers on P21, with male and female offspring separated and housed undisturbed in cages containing up to 5 mice each.

### 2.3 Experimental Design and Melatonin Treatment

**FIGURE 1:**
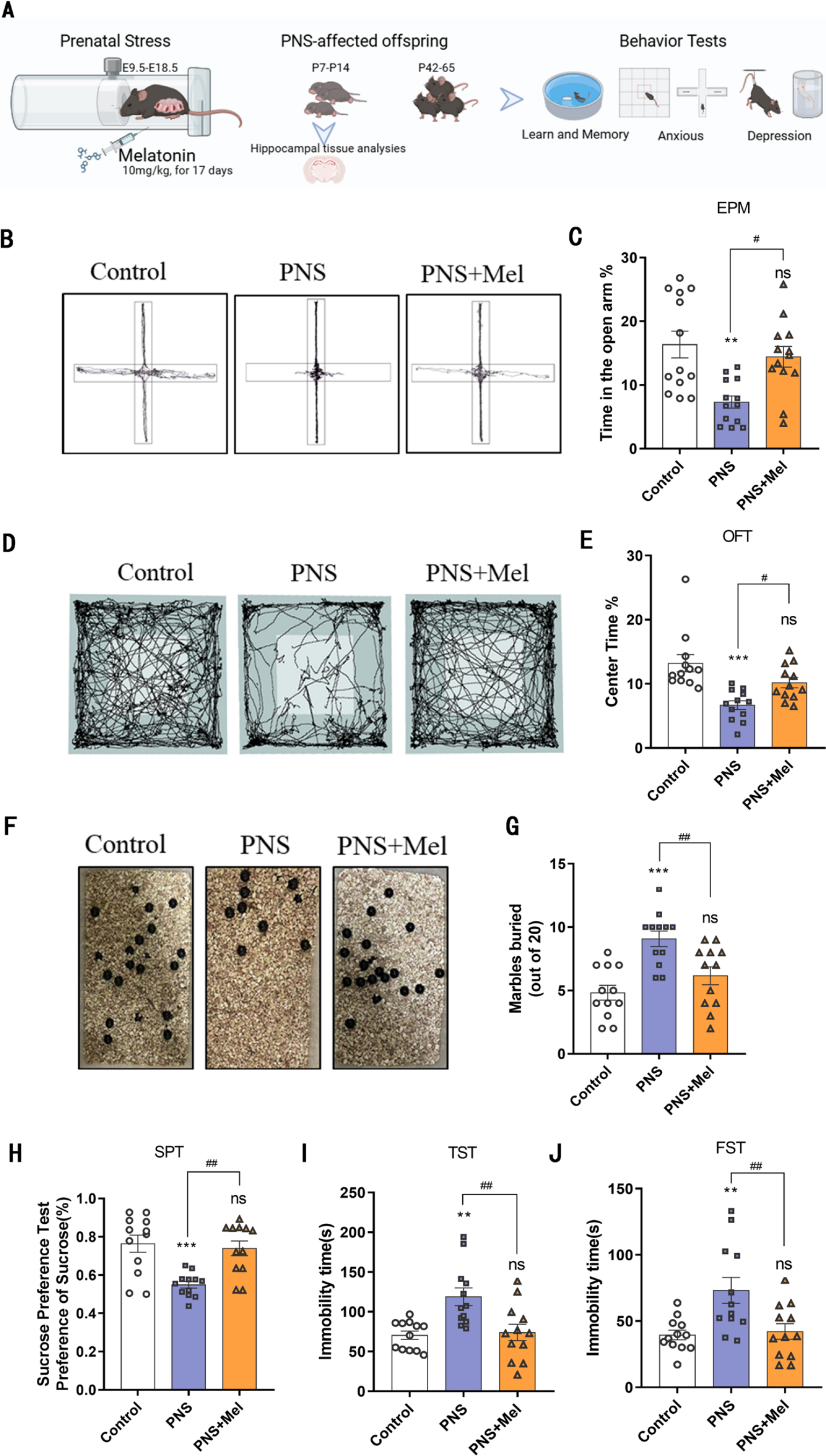
Melatonin ameliorated PNS‐induced mood disorders in offspring mice. (A) Schematic representation of the experimental design. (B) Representative trajectory of mice in EPM. (C) The duration (%) of stay in the open arm region in EPM. (D) Representative trajectory of mice in OFT. (E) The percentage of time spent in the central region of OFT. (F, G) Representative images and analysis of Marbles buried test. (H) Effect of different treatment groups on sucrose solution preference (%). (I) The immobility time of different treatment groups in TST. (J) The immobility time of different treatment groups in FST. *n* = 12 for each group. The results are shown as the mean ± SEM and analyzed by one‐way ANOVA followed by post hoc Tukey test. **p < 0.01, ***p < 0.001, compared with the Control group. #*p* < 0.05, ##*p* < 0.01 compared with the PNS group.

The experimental design and drug treatment schedule are illustrated in Figure 1A. Hippocampal samples from the mice were collected at P7 and P14, which are key stages in the neurodevelopment of the offspring, followed by neurochemical assessments. Behavioral testing began at P42. Injection of either vehicle or melatonin began at E9.5. Melatonin (Sigma‐Aldrich, USA) was dissolved in a sterile saline solution containing 1% ethanol (Solarbio, China) and administered daily for 17 days at a dose of 10 mg/kg at 4:00 p.m. (2 h before the onset of the dark phase), following established protocols from previous studies [47, 48, 49]. The Control and matched PNS groups received vehicle treatment.

### 2.4 Measurement of PNS Model

#### 2.4.1 Body Weight

Dams with vehicle/Melatonin treatment were weighed for 10 days starting from the first day of treatment (E9.5).

#### 2.4.2 Plasma Extraction

After the termination of prenatal stress, 0.1 mL of blood was collected from the tail vein of the pregnant mice, and the blood was added to an EP tube containing disodium EDTA as an anticoagulant. The mixture was then centrifuged at 8000 rpm at 4°C for 10 min. After centrifugation, the supernatant was carefully aspirated and promptly frozen at −80°C for subsequent analysis.

#### 2.4.3 Measurement of Plasma Corticosterone Concentration

Corticosterone (ng/mL) was measured by Elisa test kit according to the provided instructions of the manufacturing kit (Coat‐A‐Count, Diagnostics Products Corporation).

#### 2.4.4 Measurement of Plasma Melatonin Concentration

Melatonin (pg/mL) was measured by an Elisa test kit according to the provided instructions of the manufacturing kit (Coat‐A‐Count, Diagnostics Products Corporation).

#### 2.4.5 Reproductive Outcomes and Offspring Data Collection

We collected data on reproductive outcomes and offspring characteristics, including litter size, the sex distribution of the pups, and their weight as well as changes in weight over time. Detailed information is provided in Table 1.

**TABLE 1:** Summary of data on litter size, sex distribution of pups, body weight, and weight changes.

Group of dams(*n* = 7)	Litter size(Mean ± SD)	No. of males and females	Body weight of pups (P0，P7)
Control	8.20 ± 1.32	Males = 4.9 ± 1.66 Females = 3.3 ± 2.00	P0 = 1.13 ± 0.18 g P7 = 4.06 ± 0.70 g
PNS	7.90 ± 0.99	Males = 4.1 ± 1.79 Females = 3.8 ± 2.20	P0 = 1.06 ± 0.15 g P7 = 3.93 ± 0.32 g
PNS + Mel	8.00 ± 1.15	Males = 4.5 ± 1.18 Females = 3.5 ± 1.84	P0 = 1.1 ± 0.18 g P7 = 3.95 ± 0.53 g

*Note:* One‐way ANOVA analysis revealed no significant differences between the groups in litter size and body weight changes *p* > 0.05.

### 2.5 Measurement of Offspring's Behaviors

Behavioral tests of mice were conducted starting from P42, including 18 male and 18 female mice: Control group (*n* = 6 male and 6 female), PNS (PNS; *n* = 6 male and 6 female), and PNS + Mel (PIA; *n* = 6 male and 6 female). Each behavioral experiment was performed in an individual dark chamber, free from unwanted external light and sound. During the experiment, the operator was blinded to the treatment groups of the mice. Mice were acclimated to the chamber for at least 30 min before the experiment began. After each experiment, the behavioral device was cleaned with 75% ethanol. Mice rested for 24 h between experiments. A tracking system (TopScan Version 3.0) was used to record, measure, and analyze all behavioral experiments.

#### 2.5.1 Morris Water Maze(MWM)

MWM is a classic behavioral test widely used to assess spatial learning and memory in mice or rats, particularly related to hippocampal‐dependent spatial memory. The water maze is a circular pool with a diameter of 120 cm, filled with water maintained at a temperature of 23°C ± 2°C and supplemented with titanium dioxide. Distinct shapes are posted along the walls of the pool to serve as spatial reference cues. A camera is installed above the water maze to capture the movement trajectories of the mice.

During the training phase, a platform was submerged 1.5 cm below the water surface, and the mice were introduced into the maze facing one of the four cardinal points (N, S, E, W). Mice were allowed to search for the platform for 60 s. If the mice failed to find the platform, they were guided to it and allowed to remain there for 30 s. Four trials were performed each day with at least 1 h between them. The escape latency, indicating spatial memory acquisition, was recorded for each trial. On the 6th day, testing began, and the platform was removed. The percentage of time spent in each of the four quadrants, along with the number of crossings into the target (platform) area, average velocity, and total distance traveled, were recorded [50].

#### 2.5.2 Novel Object Recognition(NOR)

The Novel Object Recognition (NOR) test is commonly used to assess memory and cognitive function in mice or rats, particularly to measure working memory and long‐term memory. The novel object recognition test was carried out in an open field facility. On the first day, the mice underwent a 10‐min acclimation period to familiarize themselves with the environment. The next day, two identical objects were introduced into the open field, allowing the mice to observe them for a five‐minute period. On the third day, the mice were again exposed to the objects for 5 min. For the Object Recognition Memory (ORM) task, one of the familiar objects was replaced with a novel object while maintaining the original location. For the Object Location Memory (OLM) task, the mice encountered two familiar objects, with one of them relocated to a new position within the open field. Exploration time was recorded when the mice approached within 1 cm of the object or when their nose touched the object. During the test phase, the time spent by the animal exploring the new and old objects is recorded. Generally, animals tend to spend more time exploring new objects. If the animal remembers the old object, it will show more interest in the new object [51].

#### 2.5.3 Elevated Plus Maze (EPM)

The Elevated Plus Maze is a widely accepted method for measuring anxiety‐like behavior in rodents. The elevated plus maze is positioned 50 cm above the ground and comprises two open arms and two closed arms. The open arms measure 40 × 5 cm and are bordered by a 0.5 cm high edge, whereas the closed arms measure 40 × 5 cm and are enclosed by 19 cm high walls. Mice were placed on the maze's central platform (5 × 5 cm), facing an open arm, and allowed to explore the maze for 6 min. Motion data were collected using a video tracking system. The measured motion parameters included time spent in the open arms, time spent in the closed arms, time spent on the central platform, percentage of entries into the open arms, and total distance traveled. Mice exhibiting higher levels of anxiety were more likely to remain in the closed arms. Mice with higher levels of anxiety are more likely to stay in the enclosed arms of the maze [52].

#### 2.5.4 The Marble Burying Test

The Marble Burying Test is a standard experimental method widely used to assess anxiety and compulsive‐like behaviors in animals. The mice were placed individually in a cage (40 cm × 25 cm × 20 cm) with a 5 cm thick layer of corncob bedding. Each cage contained 20 glass marbles, each with a diameter of 1.5 cm, arranged in a “4 × 5” format. The number of marbles buried within 10 min was recorded. A marble was considered buried if 2/3 or more of its volume was covered by the corncob bedding. Mice with higher anxiety levels typically exhibit more marble burying behavior, indicating a strong avoidance or suppression response to novel stimuli [53].

#### 2.5.5 Open Field Test (OFT)

The Open Field Test is used to assess general locomotor activity and anxiety. The mice were placed in a 40 × 40 cm field and tested for 10 min. Initially, they were placed in the center, defined as a 20 × 20 cm area. The activity of the mice was recorded by an overhead camera. An animal behavior tracking system was used to analyze the total distance traveled and the time spent in the center. In the case of anxiety, rodents typically spend less time in the center of the arena [54].

#### 2.5.6 Tail Suspension Test (TST)

This test is commonly used to assess behavioral despair and has been validated in various rodent depression models. The mice were suspended upside down by their tails for 6 min and monitored using a video tracking system. Escape behavior was assessed, and the duration of immobility within the 6 min was recorded. Immobility was defined as the period during which a mouse exhibited no significant movement or struggling. The duration of immobility is considered a reliable indicator of depressive behavior [55].

#### 2.5.7 Forced Swim Test (FST)

The forced swim test is a well‐established method for assessing despair behavior in mice. It was performed in a glass container with a height of 30 cm and a width of 20 cm. Fresh water at a temperature of 23°C ± 2°C was filled in this container up to a depth of 20 cm. Over a period of 6 min, the duration of immobility of the mice was recorded. Immobility was defined as the time during which a mouse makes only the minimal movements necessary to keep its head above the water surface. The duration of immobility is considered a reliable indicator of depressive behavior [56].

#### 2.5.8 Sucrose Preference Test (SPT)

The sucrose preference test is based on the animal's natural preference for sweetness and is used to measure the animal's choice between water and a sucrose solution under different conditions. It is commonly used to assess the animal's interest, reward system, and depressive symptoms. The mice were housed individually. On the first day, two sources of ordinary drinking water were provided to observe any location preference. On the second day, the mice were deprived of water and food for 12 h. On the third day, one of the water sources was replaced with sucrose water, and its location was switched after 1 h. The sucrose preference rate was calculated based on the liquid consumption records of each mouse within 2 h. The sucrose preference rate (%) was calculated as follows: (sucrose liquid consumption/total liquid consumption) × 100%. The higher the ratio, the stronger the animal's preference for sweetness, indicating the normal function of its reward system. In depressed animals, the intake of sucrose solution is typically significantly lower than that of the control group, reflecting depressive‐like behavior and dysfunction of the reward system [57].

### 2.6 Immunofluorescence

Brain tissue slices or cell slides fixed with 4% paraformaldehyde were washed three times with PBS. They were then blocked with a mixture of 10% donkey serum and 0.5% Triton X‐100 (Beyotime, China) for 2 h, followed by overnight incubation with primary antibodies at 4°C. The primary antibodies used were anti‐Ki67 (1:500, CST), anti‐DCX (1:500, Abcam), anti‐Ctip2 (1:200, Abcam), anti‐Synaptophysin (1:1000, Proteintech), anti‐Iba1 (1:500, Wako), anti‐CD68 (1:500, CST), anti‐CXCL9 (1:500, Proteintech) and anti‐CXCL10 (1:500, Proteintech). After washing three times with 0.1% PBST, the slices or cells were incubated with fluorescence‐conjugated secondary antibodies (AbBkine‐488 and AbBkine‐594) for 1 h. DAPI (Sigma‐Aldrich, USA) staining was performed for 30 min. After three washes with 0.1% PBST, the slices were mounted with anti‐fade mounting medium. Images were acquired using an IX71 Olympus fluorescence microscope.

### 2.7 Nissl Staining

Frozen sections of mouse hippocampal tissue were removed from −20°C, equilibrated to room temperature for 20 min, and washed with PBS for 20 min. Liquid A and Liquid B from the toluidine blue kit (Beyotime, China) were mixed in a 1:1 ratio and incubated at 37°C for 30 min. The sections were then dehydrated using a gradient series of ethanol (70%, 80%, 90%, and 100%), dried, and sealed with neutral gum. Images were acquired using an IX71 Olympus fluorescence microscope.

### 2.8 Western Blot

Mouse hippocampal tissue or cells were lysed in RIPA buffer at 4°C for 30 min. After centrifugation, the supernatant was collected, and the total protein concentration was determined using the BCA assay kit (Beyotime, China). Equal amounts of protein from each sample were separated by 8%–15% SDS‐PAGE gel electrophoresis and transferred onto PVDF membranes. The membranes were blocked with TBST buffer containing 5% milk and incubated with primary antibodies overnight at 4°C. The secondary antibodies used were either goat anti‐mouse IgG or rabbit anti‐HRP‐conjugated IgG. Finally, the protein bands were visualized using an enhanced chemiluminescence detection kit (Millipore, USA) and analyzed using Image J software. The primary antibodies were diluted as follows: rabbit anti‐Tuj1 (1:500, CST), rabbit anti‐NeuN (1:500, CST), rabbit anti‐PSD95 (1:500, Proteintech), rabbit anti‐NF200 (1:500, Proteintech), rabbit anti‐Synaptophysin (1:500, Proteintech), rabbit anti‐CXCL10 (1:500, Proteintech), rabbit anti‐p‐p65 (1:1000, CST), rabbit anti‐p65 (1:1000, CST), rabbit anti‐p‐PI3K (1:1000, Beyotime), rabbit anti‐p‐AKT (1:1000, Proteintech), rabbit anti‐AKT (1:1000, Proteintech).

### 2.9 Golgi Staining

Fresh mouse brain tissue was immersed in Golgi stain fixing solution and gently rinsed multiple times with physiological saline solution before being transferred to a centrifuge tube. The tissue was completely immersed in Golgi staining solution and left to process for 14 days. Afterward, the brain was removed, washed extensively with distilled water, and sliced into 100 μm sections using a vibrating microtome. These sections were air‐dried overnight at 4°C. Following immersion in distilled water for 5 min, they were treated with concentrated ammonia solution for 10 min, then washed again with distilled water. Subsequently, the sections underwent acidic impregnation for 45 min in darkness, followed by another wash with distilled water. Finally, the sections were mounted with glycerol gelatin. Imaging was conducted using a panoramic scanner software to identify the scanning area, and Case Viewer 2.4 software was utilized for observation and image capture.

### 2.10 Transmission Electron Microscope

Hippocampal tissue from 14‐day‐old mice was cut into 1 mm^3^ cubes and fixed in 3% glutaraldehyde at 4°C for 2 h. After fixation, the tissue was washed three times with PBS. Subsequently, the tissue was further fixed in 1% OsO_4_ at 4°C for 2 h. The tissue underwent dehydration in an ethanol gradient and was then infiltrated with 100% epoxy resin (1:3, 1:1, 3:1) for 1 h, 4 h, and 12 h, respectively. Finally, the tissue was embedded in fresh epoxy resin and polymerized at 37°C for 12 h, 45°C for 12 h, and 60°C for 1 h. Ultra‐thin sections (150 nm) were prepared and supported on grids. These sections were stained with uranyl acetate for 20 min and lead citrate for 20 min at room temperature. Images were captured using an electron microscope.

### 2.11 Sholl Analysis

Sholl analysis was employed to assess microglial morphology, providing measurements indicative of microglial activation [106]. The original data were processed and analyzed using ImageJ. During the Sholl analysis, measurements including maximum branch length, number of branches, and number of intersections per radius (increased by 5 μm) were recorded.

### 2.12 RNA‐Seq and Data Analysis

Total RNA extracted from hippocampal tissues of the Control, PNS, and PNS + Mel groups (*n* = 3 per group) underwent RNA‐seq analysis. mRNA library preparation and transcriptome sequencing were conducted at the BGI Institute of Life Sciences in Shenzhen, China, using the BGISEQ‐500 platform. The bioinformatics workflow included data filtering, differential gene expression analysis, heatmap generation, and pathway analysis using the Kyoto Encyclopedia of Genes and Genomes (KEGG). Differentially expressed genes (DEGs) were identified using DEGseq software. This study defined the genes with FC > 1.5 and *p* ≤ 0.05 as DEGs.

### 2.13 RNA Isolation and Real‐Time Quantitative PCR

Mouse hippocampal tissues were utilized to extract total RNA employing TRIZOL reagent (Invitrogen). The purity and concentration of the total RNA were subsequently assessed using a spectrophotometer. Following this, cDNA was synthesized employing the RevertAid First Strand cDNA Synthesis Kit (Thermo Fisher Scientific). Real‐time PCR was conducted utilizing the SYBR Green Real‐Time PCR Master Mix (TOYOBO CO. Ltd., Japan). β‐actin expression was employed as the normalized control, and alterations in gene expression were calculated utilizing the 2^−ΔΔCT^ method. The primer sequences are detailed in Table 2.

**TABLE 2:** The sequences of primers.

Gene	Forward (5′‐3′)	Reverse (3′‐5′)
β‐Actin	Forward	CGTTGACATCCGTAAAGACCTC
	Reverse	CCACCGATCCACACAGAGTAC
CXCL9	Forward	TCCTTTTGGGCATCATCTTCC
	Reverse	TTTGTAGTGGATCGTGCCTCG
CXCL10	Forward	CCAAGTGCTGCCGTCATTTTC
	Reverse	GGCTCGCAGGGATGATTTCAA
MET	Forward	GTGAACATGAAGTATCAGCTCCC
	Reverse	TGTAGTTTGTGGCTCCGAGAT
SPP1	Forward	AGCAAGAAACTCTTCCAAGCAA
	Reverse	GTGAGATTCGTCAGATTCATCCG
CCL27a	Forward	ACATGTCGCGATTGAGGAGA
	Reverse	AGGCAAGGCTTCTTGCTTCT
CCL21a	Forward	GTGATGGAGGGGGTCAGGA
	Reverse	GGGATGGGACAGCCTAAACT

### 2.14 Cell Counting Kit‐8 (CCK‐8) Assay

The Cell Counting Kit‐8 (CCK‐8) assay was utilized to assess the effects of drugs on cell viability. Cells were seeded in a 96‐well plate at a density of approximately 5 × 10^3^ cells per well. The cells were treated with various concentrations of drugs for 24 h at 37°C. Subsequently, 100 μL of CCK‐8 solution (APExBIO, USA) was added to each well, followed by incubation in a CO_2_ incubator at 37 °C for 2 h. The absorbance of each well was measured at a wavelength of 450 nm using a spectrophotometer.

### 2.15 Primary Microglia Isolation and Cell Culture

Neonatal mice were sacrificed on postnatal Day 3 (P3), and their brains were dissected to remove the cerebellum, olfactory bulb, hippocampus, and meninges. The remaining brain tissue was digested using 0.25% trypsin to obtain a single‐cell suspension of mixed glial cells. The cells were then resuspended in Dulbecco's Modified Eagle Medium (Gibco, USA) supplemented with 10% fetal bovine serum (Gibco, USA) and 1% Penicillin/Streptomycin/Amphotericin B (Macgene, China; 100X). Subsequently, the cells were seeded into poly‐lysine‐coated culture flasks at a density of approximately 5 × 10^6^ cells/mL. On Day 7, floating microglia were harvested by gently tapping the culture flasks. The isolated primary microglia were then seeded into either 6‐well plates or 96‐well plates for subsequent experiments. The cells were cultured in a cell culture incubator at 37°C with 5% CO_2_.

### 2.16 Microglia Phagocytosis Assay

Primary microglia were plated into 12‐well plates at a density of 5 × 10^5^ cells/mL. After pretreatment with melatonin for 4 h followed by stimulation with corticosterone for 24 h, the cell medium was replaced with DMEM and incubated in a cell culture incubator at 37°C for 30 min. Fluorescent latex beads (Sigma‐Aldrich, USA) were preconditioned by mixing with 50% FBS and PBS. Subsequently, the prepared beads were added to the cells at a concentration of 75 beads per cell and incubated at 37°C for 2 h. After incubation, the remaining beads adhered to the cells were washed away. The cells were then fixed with 4% paraformaldehyde for 30 min and stained with DAPI for 20 min. Finally, images were acquired using an IX71 Olympus fluorescence microscope.

### 2.17 N2a Cell Culture and Neurite Measurement

Mouse neuroblastoma cells (N2a cells, ATCC, USA) were cultured in Minimum Essential Medium (MEM, Gibco, USA) supplemented with 10% BS (Gibco, USA) and 1% penicillin/streptomycin (Macgene, China). Cells were incubated at 37°C with 5% CO_2_ and seeded at a density of 5 × 10^4^ cells per well in 12‐well plates.

For the experiment investigating the effect of microglial treatment on neurite growth, microglia were treated for 24 h under different conditions, including the following treatment groups: solvent control, melatonin group (1 μM), corticosterone group (50 μM), and melatonin + corticosterone combination group (1 μM melatonin and 50 μM corticosterone). After 24 h, the conditioned medium from microglia was collected and applied to N2a cells for coculture.

For the experiment investigating the effect of CXCL10 receptor antagonist on neurite growth, microglia were treated for 24 h under different conditions, including the solvent control and corticosterone group (50 μM). After 24 h, the conditioned medium from treated microglia was collected and used for coculture with N2a cells. To investigate the role of CXCL10, N2a cells were pre‐treated with CXCR3 receptor antagonist AMG487 (10 nM, Selleck, USA) for 30 min prior to coculture. The experimental groups were: Control group (N2a cells cocultured with normal microglial conditioned medium), AMG487 group (N2a cells pre‐treated with AMG487 and cocultured with normal microglial conditioned medium), CORT group (N2a cells cocultured with corticosterone‐treated microglial conditioned medium), and CORT + AMG487 group (N2a cells pre‐treated with AMG487 and cocultured with corticosterone‐treated microglial conditioned medium).

After coculturing N2a cells with microglial conditioned medium under the specified treatment conditions, the cells were further cultured in medium containing 20 μM retinoic acid (RA, MCE, USA) and 1% FBS for 48 h to induce neurite outgrowth. Neurite growth was observed under a microscope, and the length of the neurites was measured using ImageJ software. The average neurite length from multiple fields of view was quantified to represent the extent of neurite growth in each experimental group.

### 2.18 Statistical Analysis

The data were presented as the mean ± standard error of the mean (SEM). The Shapiro–Wilk test was used to test for normality. Statistical analysis involved one‐way analysis of variance (ANOVA) followed by Tukey's post hoc test. A *p*‐value less than 0.05 was considered statistically significant.

## 3 Results

### 3.1 Melatonin Improved Behavior Abnormalities in Offspring Mice Induced by PNS

The schematic diagram of the experimental design is shown in Figure 1A. During the restraint stress period, we monitored the weight of pregnant mice. Mice in the PNS group showed significantly reduced weight gain, whereas the PNS+Mel group exhibited a weight gain pattern similar to the control group (Figure S1A). Behavioral tests on the pregnant mice revealed that those in the PNS group had decreased sucrose preference and increased immobility in forced swim and tail suspension tests, suggesting depressive‐like symptoms. However, the PNS+Mel group showed improvement in these behaviors (Figure S1B,C). Plasma assays revealed increased corticosterone and decreased melatonin levels in the PNS group. Melatonin treatment effectively mitigated the rise in corticosterone and maintained melatonin levels in the PNS+Mel group (Figure S1D,E). To detect the effect of melatonin on abnormal emotional behavior in the offspring mice, we used the elevated plus maze, open field test, and marble burying test to evaluate anxiety‐like behavior. Sucrose preference tests, tail suspension tests, and forced swimming tests were employed to assess depression‐like behavior in the offspring. The results showed that, compared with the control group, the time spent in the open arms of the elevated plus maze was significantly reduced in the PNS group, while it was significantly increased in the PNS+Mel group (Figure 1B,C). Open‐field experiments indicated that the time spent in the central region was significantly reduced in the PNS group, but significantly increased in the PNS+Mel group (Figure 1D,E). In the marble burying test, the PNS group buried more marbles within the same time period compared to the control group, whereas the PNS+Mel group buried fewer marbles (Figure 1F,G). In the sucrose preference test, PNS group mice showed a lower sucrose preference rate compared to the control group, while the sucrose preference rate in the PNS+Mel group increased compared to the PNS group (Figure 1H). Tail suspension and forced swimming tests showed increased immobility time in PNS group mice, while melatonin treatment significantly improved these abnormal behaviors (Figure 1I,J).

Furthermore, prenatal stress is implicated in cognitive decline in offspring. To assess this, we evaluated the learning and memory abilities of offspring mice using the Morris water maze and novel object recognition tests. The results indicated that offspring exposed to prenatal stress exhibited significantly longer latency periods in the Morris water maze task and a reduced number of crossings into the target quadrant in the spatial exploration test. However, melatonin treatment significantly shortened the latency period in the Morris water maze task and increased the number of crossings into the target quadrant, indicating a marked improvement in learning and memory abilities (Figure S2A–E). The novel object recognition test comprises two components: object recognition memory (ORM) and object location memory (OLM). Results showed no significant differences among the three groups of mice in the ORM task (Figure S2H). In contrast, in the OLM task, mice in the PNS group exhibited significant memory deficits, while melatonin‐treated mice showed substantial improvement, approaching levels observed in the control group (Figure S2I). This suggests that melatonin treatment effectively mitigates spatial memory deficits induced by prenatal stress in offspring.

In summary, these findings suggest that melatonin treatment may alleviate the adverse effects of prenatal stress on anxiety, depression‐like behaviors, and learning and memory abilities in offspring mice.

### 3.2 Melatonin Protects Against Impaired Hippocampal Neurogenesis Caused by Prenatal Stress

**FIGURE 2:**
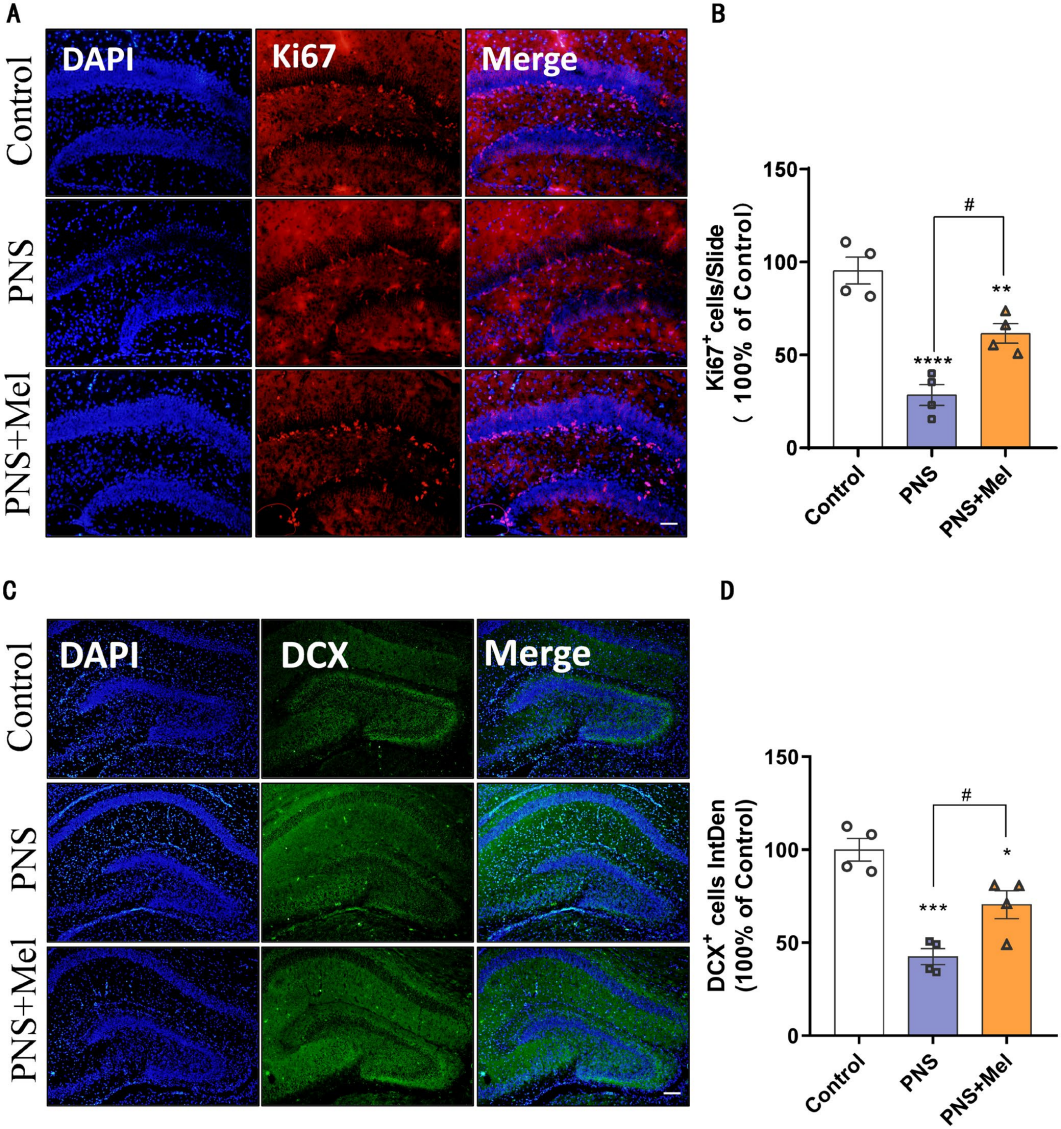
Melatonin protects against impaired hippocampal neurogenesis caused by prenatal stress. (A, B) Representative images and quantification of immunofluorescence analysis of Ki67^+^ cells in different groups of hippocampal DG. Scale bars: 50 μm. (C, D) Representative images and quantification of immunofluorescence analysis of DCX^+^ cells in different groups of hippocampal DG. Scale bars: 100 μm. *n* = 4 for each group. The results are shown as the mean ± SEM and analyzed by one‐way ANOVA followed by post hoc Turkey test. *p < 0.05, **p < 0.01, ***p < 0.001, ****p < 0.0001 compared with the Control group. #*p* < 0.05 compared with the PNS group.

Ki67 and DCX staining were conducted on postnatal day 7 (P7) mice. The results indicate that prenatal stress significantly reduced the number of Ki67^+^ and DCX^+^ cells in the dentate gyrus of newborn mice compared to the control group, while melatonin rescued the reduction caused by prenatal stress (Figure 2A–D). Specifically, prenatal stress decreased the number of newborn neurons in the hippocampal dentate gyrus of offspring, while melatonin treatment mitigated impaired hippocampal neurogenesis.

### 3.3 Melatonin Improves the Reduction of Hippocampal Neurons and Synaptic Defects Caused by PNS

**FIGURE 3:**
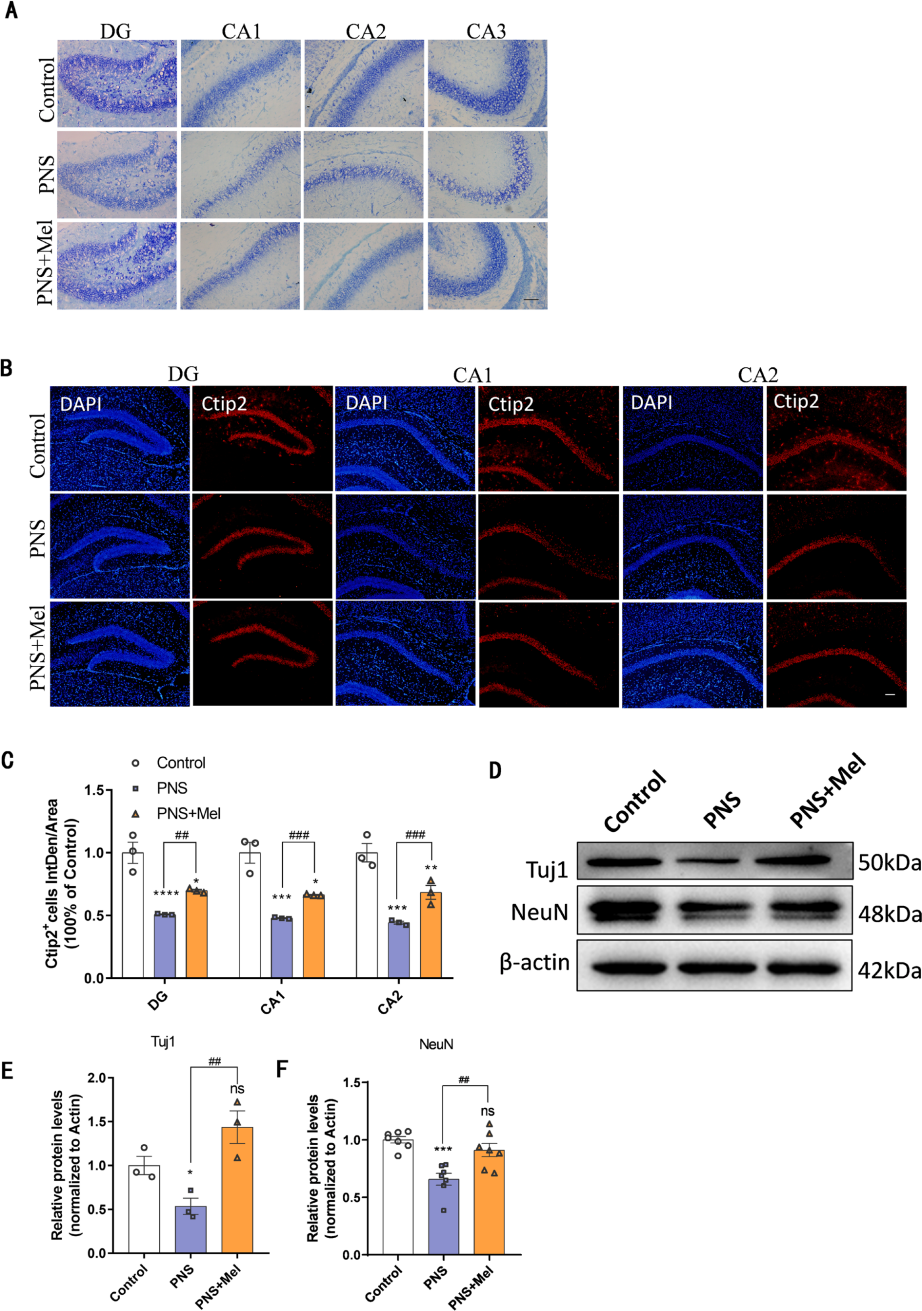
Melatonin improves the number of hippocampal neurons decreased by PNS. A Representative images of the Nissl staining of the hippocampus in different groups. Scale bar: 100 μm. B‐C Representative images and quantification of immunofluorescence analysis of Ctip2^+^ cells in different groups of the hippocampus. Scale bars: 100 μm. D‐F Representative western blot images and relative quantification of Tuj1 and NeuN. *n* = 3–7 for each group. The results are shown as the mean ± SEM, and analyzed by one‐way ANOVA followed by post hoc Tukey test. *p < 0.05, **p < 0.01, ***p < 0.001, ****p < 0.0001 compared with the Control group. ##*p* < 0.01, ###*p* < 0.001, compared with the PNS group.

Next, we examined the effect of melatonin on neuronal maturation in hippocampal tissue from offspring at 14 days postnatal. Nissl staining revealed that, compared to the control group, the PNS group exhibited fewer hippocampal neurons and reduced Nissl bodies. In contrast, the neuronal status in the PNS+Mel group resembled that of the control group (Figure 3A). Granule cells are a predominant cell type in hippocampal tissue, with Ctip2 serving as a marker for these neurons. Immunofluorescence staining indicated a decrease in granule neurons in the PNS group compared to the control group, while the PNS+Mel group showed an increase in granule neuron numbers compared to the PNS group (Figure 3B,C). NeuN and Tuj1, widely used neuronal markers, were assessed via Western blot. Results indicated that prenatal stress reduced the expression of NeuN and Tuj1 in the hippocampus of offspring mice compared to the control group, whereas expression levels were increased in the PNS+Mel group compared to the PNS group (Figure 3D–F).

**FIGURE 4:**
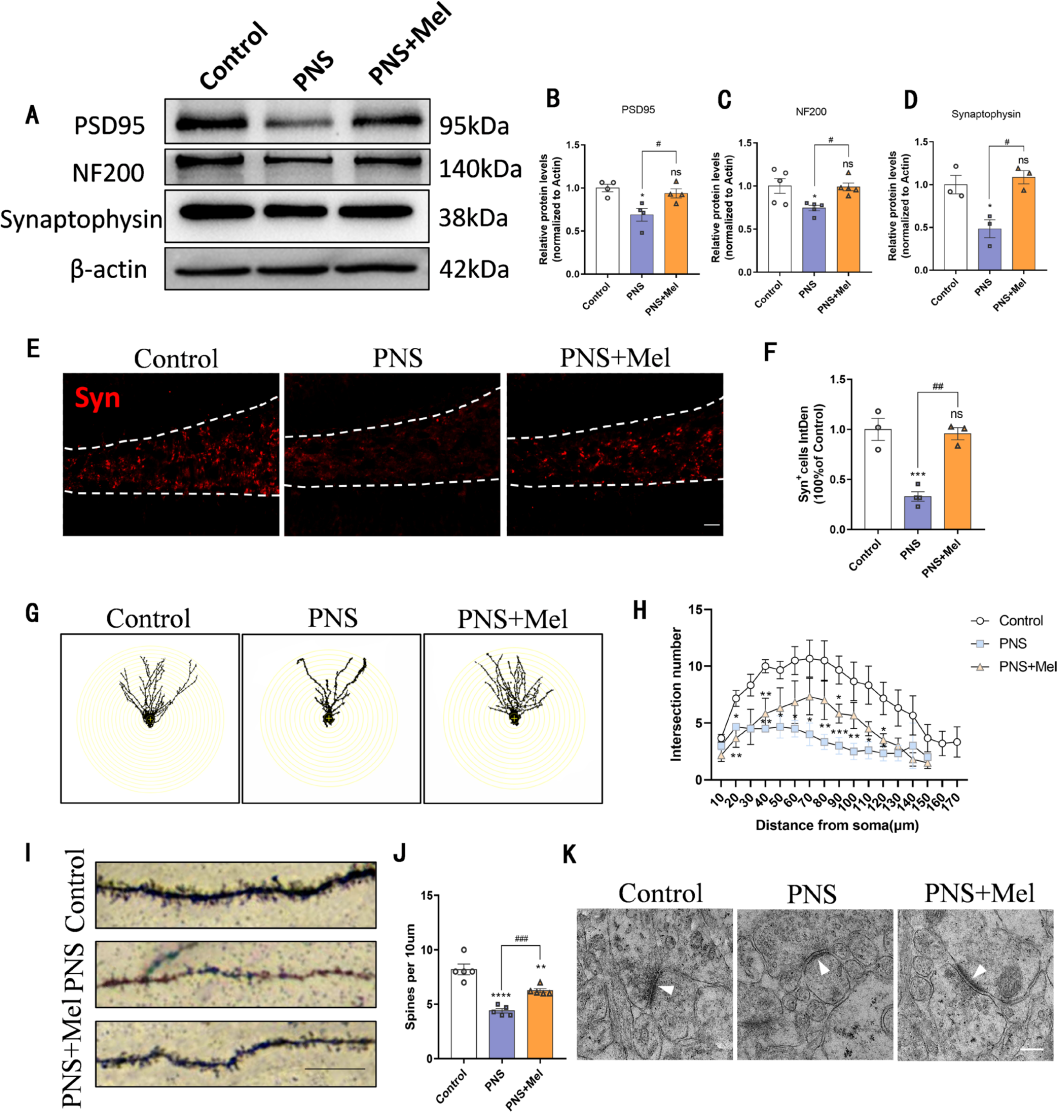
Melatonin improves hippocampal synaptic defects caused by PNS. (A–C) Representative western blot images and relative quantification of PSD95, NF200, and Synaptophysin. (D, E) Representative images and quantification of immunofluorescence analysis of Synaptophysin expression in different groups of DG. Scale bars: 50 μm. (F‐J) Representative images and analysis of Golgi staining of hippocampus. Scale bar: 20 μm. K Representative images of synapsis ultrastructure. Scale bar: 500 nm. *n* = 3–6 for each group. The results are shown as the mean ± SEM and analyzed by one‐way ANOVA followed by post hoc Tukey test. *p < 0.05, **p < 0.01, ***p < 0.001, ****p < 0.0001 compared with the Control group. #*p* < 0.05, ##*p* < 0.01, ###*p* < 0.001  compared with the PNS group.

In rodents, the critical period for hippocampal synaptic formation occurs 2–3 weeks after birth. Western blot analysis revealed that compared with controls, decreased expression of synaptic‐related proteins Syn, NF200, and PSD95 was found in the hippocampus of mice subjected to prenatal stress, whereas their levels were elevated in the PNS+Mel group relative to the PNS group (Figure 4A–D). Immunofluorescence staining indicated reduced optical density of Syn protein expression in the dentate gyrus of prenatal stress‐exposed mice compared to controls, with higher density observed in the PNS+Mel group relative to the PNS group (Figure 4E,F). We used Golgi staining to examine the dendrites and spines of hippocampal neurons in the brain. Sholl analysis revealed that the neuronal dendrites in the hippocampal dentate gyrus region of mice in the PNS group were less complex than those in the control group. However, the dendrites in the PNS+Mel group exhibited greater complexity compared to the PNS group (Figure 4G,H). Additionally, the density of hippocampal neuronal dendritic spines was reduced in the PNS group relative to the control group, while the PNS+Mel group demonstrated an increased spine density compared to the PNS group (Figure 4I,J). Furthermore, electron microscopy confirmed that melatonin mitigated the reduction in postsynaptic membrane density induced by prenatal stress (Figure 4K).

### 3.4 RNA‐Seq Analysis Reveals That Melatonin Suppresses PNS‐Induced Immune Response in the Hippocampus

**FIGURE 5:**
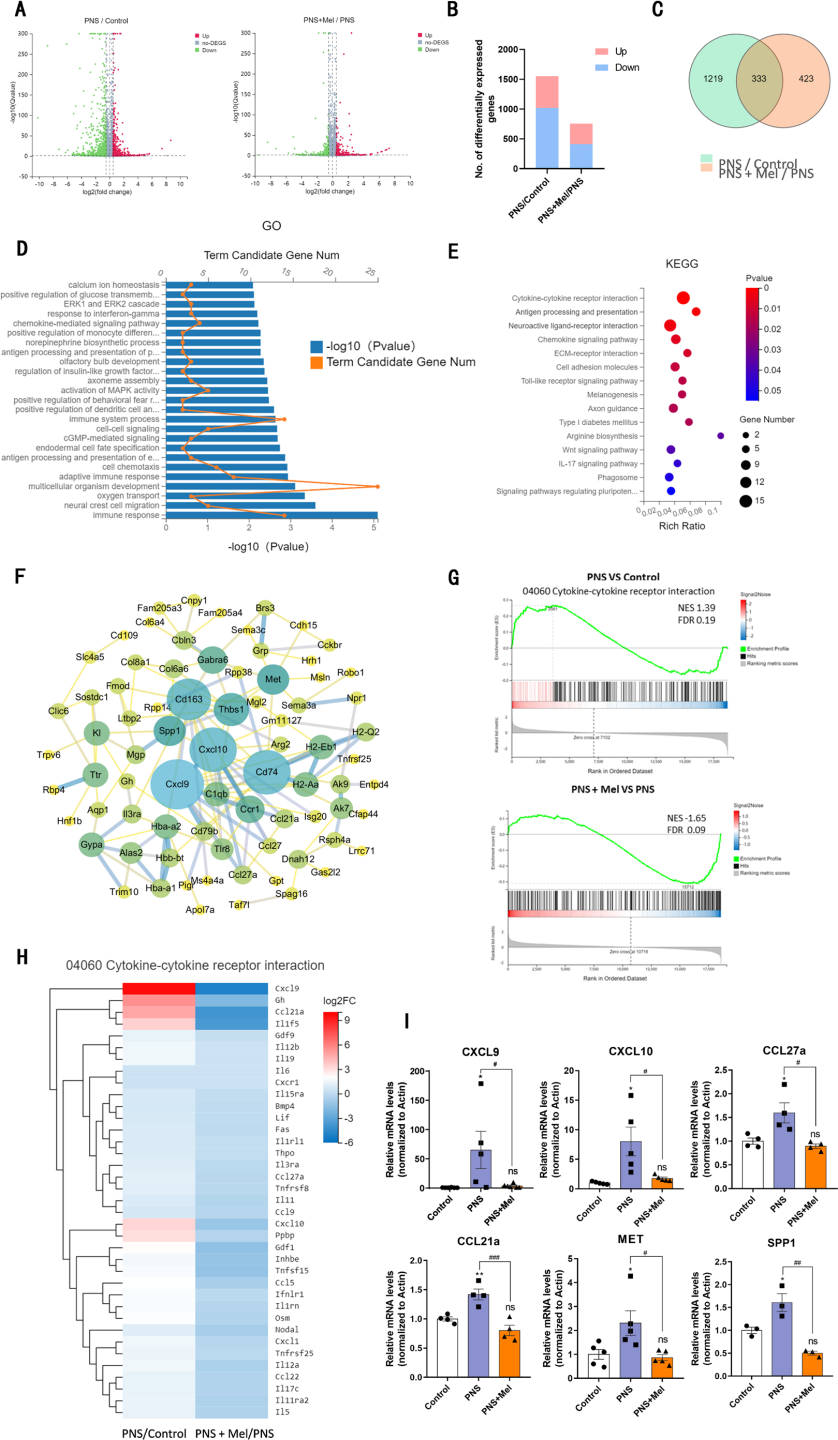
RNA‐seq analysis of hippocampal tissues from the Control, PNS, and PNS+Mel groups. (A) Volcano plot shows gene expression in the hippocampus, comparing significant increases (red) or decreases (blue) in gene expression between the control and PNS groups (left) or PNS and PNS+Mel groups (right) (*p* < 0.05, |log2|> 0.5). (B) Hierarchical clustering of all significantly altered gene expressions. Red indicates upregulated genes, and blue indicates downregulated genes. (C) The number of shared and unique DEGs in the hippocampus between the PNS and PNS+Mel groups. (D) GO biological process enrichment analysis of DEGs between the control, PNS, and PNS+Mel groups. (E) KEGG enrichment analysis further analyzes the function of differentially expressed genes. (F) STRING analysis of the top 330 regulatory gene interactions restored after melatonin treatment. (G) GSEA indicates that melatonin treatment restores the downregulation of cytokine‐cytokine receptor interaction genes in the hippocampus induced by PNS. (H) Heatmap showing the expression of top regulatory genes in cytokine‐cytokine receptor interactions in the hippocampus of the PNS and PNS+Mel groups. (I) Compared to the PNS group, melatonin treatment significantly reduces the expression of genes related to immune regulation in the hippocampus. The results are shown as the mean ± SEM and analyzed by one‐way ANOVA followed by post hoc Turkey test. *p < 0.05, **p < 0.01, compared with the Control group. #*p* < 0.05, ##*p* < 0.01, ###*p* < 0.001 compared with the PNS group.

To further understand the molecular mechanisms by which melatonin affects the offspring of prenatal stress (PNS) exposure, we performed transcriptomic sequencing analysis on hippocampal tissues from the control, PNS, and PNS+Mel groups. Compared to the control group, 1552 DEGs were identified in the PNS group (corrected *p* < 0.05, with 531 upregulated and 1021 downregulated), and 758 DEGs were identified in the PNS+Mel group (343 upregulated and 413 downregulated) (Figure 5A,B). Further cross‐genotype comparisons indicated that melatonin treatment reversed approximately one‐fifth of the DEGs identified between the PNS and control groups (Figure 5C). To explore the functional roles regulated by melatonin, KEGG pathway analysis was conducted on the differentially expressed genes. GO analysis revealed that the biological processes enriched for target genes were mainly associated with immune response, antigen processing and presentation, neural crest cell migration, multicellular organism development, adaptive immune response, cell chemotaxis, cGMP‐mediated signaling, intercellular signal transduction, immune system processes, and regulation of insulin‐like growth factors (Figure 5D). The KEGG results indicated that the DEGs were primarily enriched in pathways including cytokine‐cytokine receptor interaction, ECM‐receptor interaction, chemokine signaling pathway, cell adhesion molecules, phagosome, arginine biosynthesis, neuroactive ligand‐receptor interaction, Toll‐like receptor signaling pathway, circadian rhythm, PI3K‐AKT signaling pathway, and axon guidance (Figure 5E). To investigate the potential mechanisms underlying the therapeutic effects of melatonin, we analyzed the known and predicted protein interactions of these differential genes. The results indicated that the central regulatory proteins among these genes included Cxcl9, Cxcl10, Cd74, Cd163, Thbs1, Spp1, and Met, all of which are related to immune responses associated with glial cell activation (Figure 5F).

GSEA demonstrated that genes associated with cytokine‐cytokine receptor interaction (rno04060) were upregulated in the PNS group compared to the control group (NES = 1.39, FDR = 0.19). Melatonin treatment significantly downregulated these genes (NES = −1.65, FDR = 0.09) (Figure 5G). To validate the reliability of the RNA‐seq and DEGs analysis, we performed qRT‐PCR to verify the expression of key significant genes, including Cxcl9, Cxcl10, Ccl27a, Ccl21a, Spp1, and Met. These genes were elevated in the hippocampus of the PNS group. Melatonin treatment significantly reduced their transcription levels, consistent with the RNA‐seq results (Figure 5H,I).

### 3.5 Melatonin Ameliorates PNS‐Induced Microglia Hyperactivation in the Hippocampal Dentate Gyrus

**FIGURE 6:**
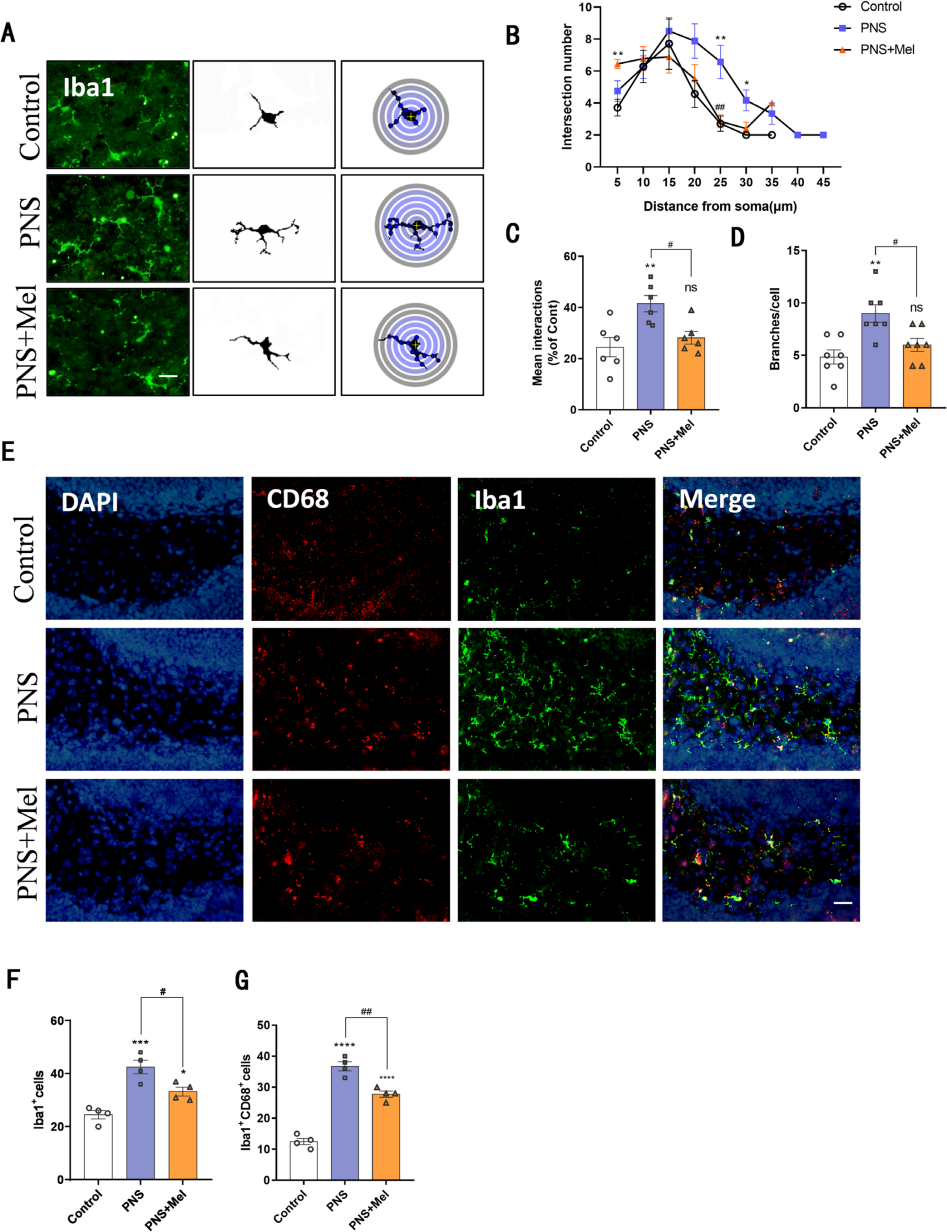
Melatonin ameliorates PNS‐induced microglia hyperactivation in the hippocampal dentate gyrus. (A–D) Sholl analysis of microglia and quantification of cell morphology in different groups of DG. Scale bars: 20 μm. (E–G) Representative images and quantification of immunofluorescence analysis of Iba1 (green)/CD68 (red) in different groups of DG. Scale bars: 20 μm. *n* = 4–8 for each group. The results are shown as the mean ± SEM and analyzed by one‐way ANOVA followed by post hoc Tukey test. *p < 0.05, **p < 0.01, ***p < 0.001, ****p < 0.0001 compared with the Control group. #*p* < 0.05, ##*p* < 0.01, compared with the PNS group.

Given that microglia are the primary immune cells in the brain and play critical roles in neurogenesis and synapse formation, we investigated the effects of melatonin on hippocampal microglia of mice on postnatal Day 14. Immunofluorescence staining of Iba1 and Sholl analysis revealed that microglia in the PNS group had longer and more branches compared to the control group, while melatonin treatment normalized the branching morphology of microglia (Figure 6A–D). Meanwhile, the results showed that at postnatal Day 14, compared to the control group, the number of microglia in the dentate gyrus of the hippocampus increased in the PNS group. Conversely, the number of microglia in the PNS+Mel group was reduced compared to the PNS group (Figure 6E,F). Double staining with CD68 and Iba1 showed that the proportion of CD68^+^Iba1^+^ microglia was higher in the PNS group, indicating a higher activation and phagocytic state of microglia. In contrast, melatonin treatment reduced the proportion of CD68^+^Iba1^+^ microglia (Figure 6G).

### 3.6 Melatonin Regulates CXCL10 Expression Through the Pl3K/AKT/NF‐κB Signaling Pathway

**FIGURE 7:**
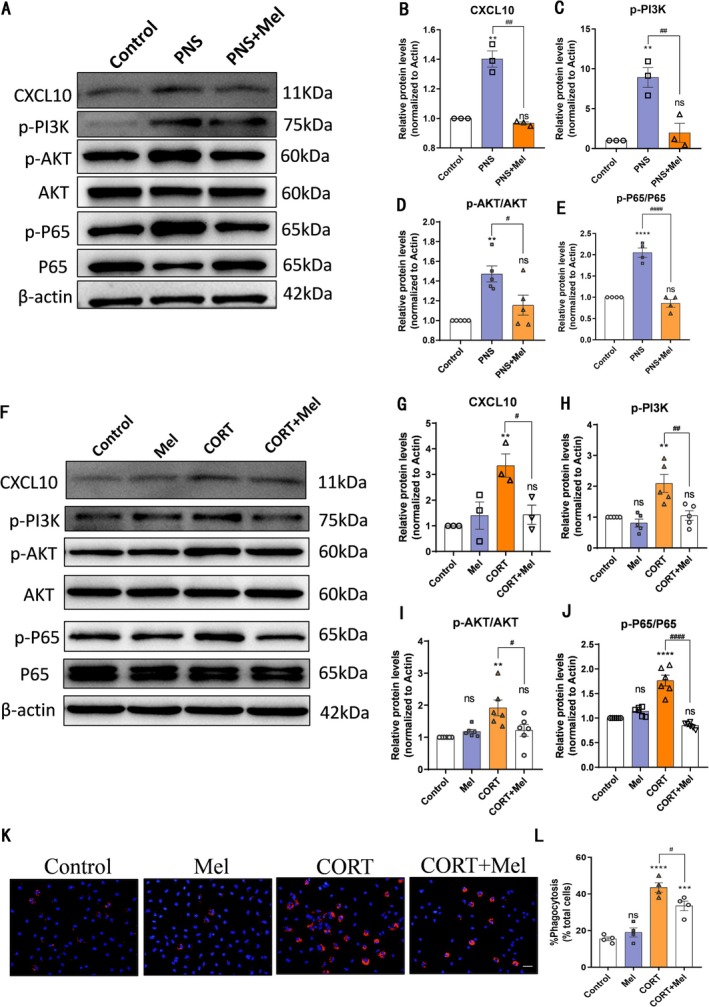
Melatonin regulates CXCL10 expression through the Pl3K/AKT/NF‐κB signaling pathway. A‐E Representative western blot images and relative quantification of CXCL10, p‐PI3K, PI3K, p‐AKT, AKT, p‐p65, P65. F‐J Representative western blot images and relative quantification of CXCL10, p‐PI3K, PI3K, p‐AKT, AKT, p‐P65, P65. K‐L Alterations in phagocytosis function of microglia were measured by Phagocytosis assay. Scale bars: 100 μm. *n* = 3–6 for each group. The results are shown as the mean ± SEM and analyzed by one‐way ANOVA followed by post hoc Turkey test. **p < 0.01, ***p < 0.001, ****p < 0.0001 compared with the Control group. #*p* < 0.05, ##*p* < 0.01, ####*p* < 0.0001 compared with the PNS group.

Transcriptomic analysis identified CXCL9 and CXCL10 as core molecules in the differentially regulated network. However, subsequent experimental validation revealed distinct characteristics between these two chemokines. For CXCL9, immunofluorescence staining demonstrated its sparse and diffuse expression in the brain, with no statistically significant differences observed between groups (Figure S3A,B). In contrast, CXCL10 exhibited robust expression in the hippocampal dentate gyrus, a region densely populated with neuronal axons and synapses (Figure S3C,D). As a gene associated with microglial polarization and inflammatory signaling, CXCL10 is known to regulate synaptic activity in hippocampal neurons [58, 59, 60, 61]. We further used Western Blot to confirm changes in CXCL10 expression, and the results indicated that CXCL10 expression was significantly increased in the PNS group but reduced in the PNS+Mel group (Figure 7A,B). We also validated that the PI3K/AKT and NF‐κB signaling pathways were activated in the hippocampus of offspring subjected to prenatal stress, whereas melatonin effectively inhibited these pathways (Figure 7C–E).

In vitro, we used corticosterone to stimulate microglia to mimic in vivo stress conditions and treated them with melatonin. To determine the optimal concentrations of corticosterone and melatonin, we performed CCK‐8 assays (Figure S4A,B). Based on the results, we selected 50 μM corticosterone and 1 μM melatonin for further experiments. Through Western blot analysis, we discovered that corticosterone treatment increased the expression of p‐PI3K, p‐AKT, and p‐P65 in microglia and induced the expression of CXCL10. However, melatonin treatment ameliorated these protein changes (Figure 7F–J). Additionally, using a fluorescent latex bead phagocytosis assay, we found that corticosterone treatment enhanced the phagocytic activity of microglia. However, melatonin inhibited the corticosterone‐induced enhancement of microglial phagocytic activity (Figure 7K,L).

### 3.7 CXCL10 Secreted by Microglia Inhibits Neurite Outgrowth In Vitro

**FIGURE 8:**
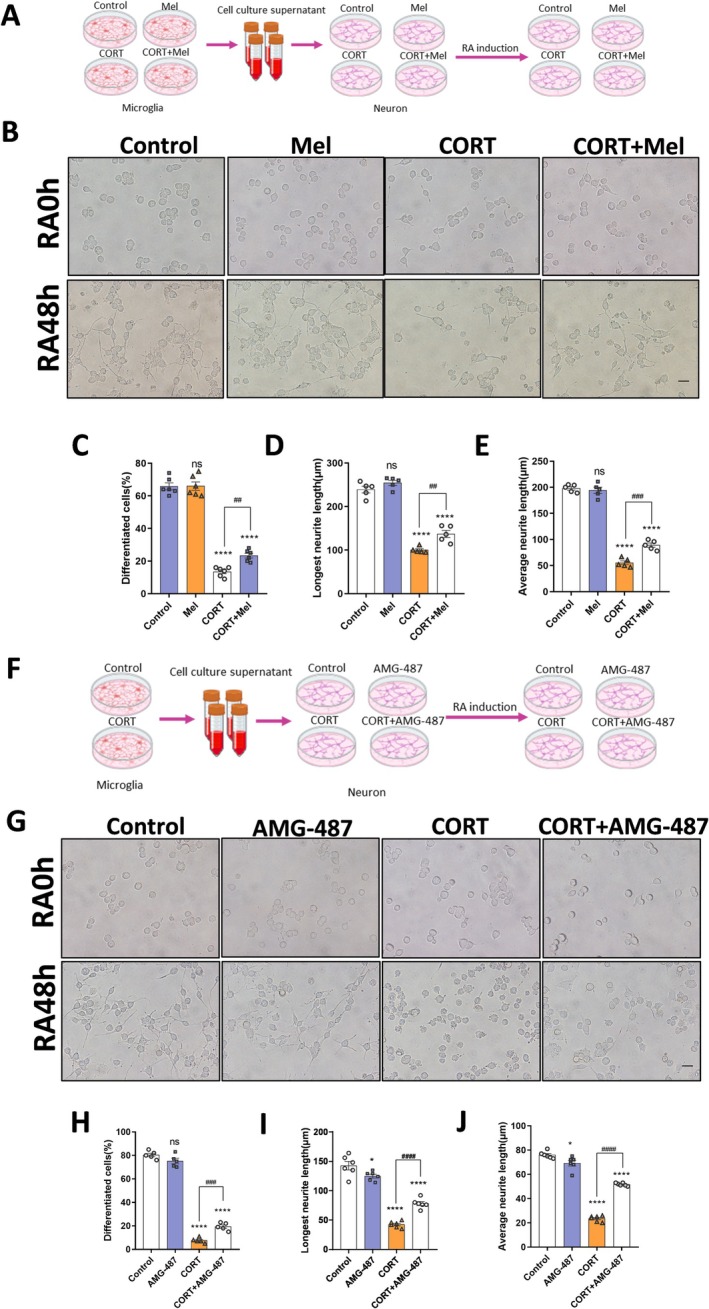
CXCL10 secreted by microglia inhibits neurite outgrowth in vitro. (A) Schematic diagram of in vitro experiment design. (B) Bright‐field images are shown for N2a cells that were treated with the supernatants of microglial cells in the Control, Mel, CORT, and CORT+Mel groups, respectively, and were induced by RA for 48 h. (C–E) The statistical analyses of the cell morphology and neurite outgrowth are indicated for the N2a cells. (F) Schematic diagram of in vitro experiment design. (G) Bright‐field images are shown for N2a cells that were treated with the supernatants of microglial cells in the Control and CORT groups, respectively, and were induced by RA for 48 h. Additionally, two of the groups of N2a cells were pretreated with AMG487. H‐J The statistical analyses of the cell morphology and neurite outgrowth are indicated for the N2a cells. *n* = 6 for each group. The results are shown as the mean ± SEM, and analyzed by one‐way ANOVA followed by post hoc Turkey test. *p < 0.05, ****p < 0.0001 compared with the Control group. ##*p* < 0.01, ###*p* < 0.001, ####*p* < 0.0001 compared with the CORT group.

After separately treating primary microglial cell cultures with vehicle solution, melatonin, corticosterone, and corticosterone combined with melatonin, we applied these conditioned media to N2a cells to simulate an in vitro coculture environment of microglia and neurons (Figure 8A). Subsequently, we induced differentiation of N2a cells with retinoic acid. We observed that, compared to the control group, conditioned media from corticosterone‐treated microglia significantly inhibited neurite outgrowth in N2a neurons (Figure 8B), reduced the number of differentiated cells (Figure 8C), decreased the longest neurite length (Figure 8D), and shortened the average neurite length (Figure 8E). There was no significant difference between the Mel group and the control group, while the CORT + Mel group partially rescued neurite outgrowth compared to the CORT group. Furthermore, to investigate the role of CXCL10 in this context, we treated N2a cells with the CXCL10 receptor CXCR3 inhibitor AMG487. To determine the optimal concentration of AMG487 and ensure its non‐toxic effects on N2a cells, we performed CCK‐8 assays (Figure S4C). Based on the results, we selected 10 nM AMG487 for further experiments. We found that AMG487 had no significant effect on neurite outgrowth in normal microglia conditioned medium compared to the control group. However, AMG487 mitigated neurite outgrowth damage induced by corticosterone‐treated microglia conditioned media (Figure 8F–J).

These experimental results suggest that prenatal stress activates microglial phagocytic function and induces CXCL10 expression. Melatonin treatment can modulate microglia through the PI3K/AKT/NF‐κB pathways, suppressing CXCL10 expression and thereby influencing neuronal development.

## 4 Discussion

Prenatal stress significantly impacts offspring development, yet effective pharmacological interventions to alleviate this stress remain lacking. Our study demonstrates that melatonin treatment improves learning, memory, and emotional disorders in offspring exposed to prenatal stress. It also enhances neurogenesis in the hippocampal dentate gyrus, counters reductions in granule neurons, and mitigates abnormalities in synaptic development in mice. Moreover, melatonin attenuates microglial cell activation and enhances their phagocytic activity, which may affect neural development. Using hippocampal tissue RNA sequencing and in vivo and in vitro validation, we have identified that melatonin inhibits the secretion of the microglial chemokine CXCL10 by suppressing the PI3K/AKT/NFκB pathways, thereby influencing neurodevelopment.

This study demonstrates that melatonin can mitigate behavioral abnormalities induced by prenatal stress in offspring. Extensive research indicates that prenatal stress consistently impacts offspring, contributing to a spectrum of issues including emotional, cognitive, interpersonal, neuroendocrine, and brain developmental challenges [62, 63, 64, 65]. Animal studies illustrate that prenatal restraint stress induces depression‐like behaviors in both male and female rodents [66], and cognitive deficits have been observed in rodent offspring exposed to maternal stress [67]. Various stress models have highlighted the beneficial effects of melatonin on learning, memory, and mood. For example, daily administration of 10 mg/kg melatonin for 3 weeks reduces corticosterone‐induced depression and anxiety‐like behaviors in mice [68]. Our study also shows that melatonin treatment effectively mitigates maternal depressive‐like behaviors induced by prenatal stress and reduces elevated plasma corticosterone levels. Additionally, melatonin may prevent memory deficits in offspring exposed to maternal dual exposure to ethanol and stress by inhibiting oxidative stress [30]. However, it is still unclear whether melatonin has an improving effect on offspring behavioral abnormalities caused by prenatal stress. In our study, employing a series of behavioral tests, maternal melatonin treatment effectively enhances learning, memory, and emotional disturbances in offspring exposed to prenatal stress, aligning with previous research findings.

The hippocampus is intricately connected with other limbic regions of the brain, including the hypothalamus, amygdala, prefrontal cortex, and nucleus accumbens [69]. Numerous neuroimaging studies have demonstrated that these specific brain regions, as well as their interconnections, play critical roles in regulating stress responses, anxiety, and the formation of emotional memories [70, 71, 72, 73, 74, 75, 76, 77, 78]. Hippocampal neurons selectively transmit behaviorally relevant information to distinct target regions, and these complex functional connections are key factors underlying the memory and emotional deficits associated with hippocampal dysfunction [69]. During hippocampal neurogenesis, neural progenitor cells progressively differentiate into neurons and interneurons, which integrate into existing neural circuits. This process provides the structural and functional plasticity essential for the hippocampus to adapt and perform its diverse roles in cognition and emotion [79]. Melatonin has been reported to alleviate anxiety, depression‐like behaviors, and enhance learning and memory through various mechanisms, including the promotion of hippocampal neurogenesis, dendritic growth, and axonal development in adults [68, 80, 81, 82, 83]. Numerous studies have demonstrated that early‐life stressors, including both prenatal and postnatal factors, exert long‐lasting effects on hippocampal neurogenesis, neuronal composition, and hippocampal plasticity. These alterations have profound implications for emotional and cognitive behaviors throughout the lifespan [84, 85, 86, 87, 88]. Our findings demonstrate that maternal melatonin treatment effectively ameliorates stress‐induced abnormalities in hippocampal neurogenesis and reduces the number of granule neurons. Western blot analysis reveals reduced expression of synaptic membrane‐related proteins in the presynaptic and postsynaptic membranes of hippocampal neurons under prenatal stress. Golgi staining indicates a decrease in dendritic spine density in mice exposed to prenatal stress, while electron microscopy suggests that prenatal stress may compromise synaptic structural plasticity, a condition melatonin counteracts. We selected P7 and P14 to capture distinct stages of neurogenesis. The connection between neurodevelopment and later behavior is not an “isolated” investigation but rather a natural scientific process. Early neurodevelopmental events can serve as potential predictors for subsequent behavioral changes. Thus, we propose that melatonin's enhancement of emotional regulation and learning and memory may stem from its role in mitigating defects in hippocampal neurogenesis, neuronal development, and synaptic plasticity. The temporal continuity of our study highlights the potential of melatonin for early intervention in mitigating hippocampal developmental abnormalities and behavioral deficits induced by prenatal stress in offspring.

Microglia are active participants in complex neurodevelopmental processes. They can phagocytize excess neural precursor cells and synapses to prevent neurodevelopmental defects while also promoting the refinement of neural circuits by secreting various brain‐derived neurotrophic factors and other growth factors [89, 90, 91]. However, under pathological conditions, abnormal activation and phagocytosis of microglia can lead to neurodevelopmental abnormalities and neurodegeneration [92, 93]. Using transcriptome sequencing, we found that melatonin therapy reduced microglial gene enrichment and up‐regulation of immune‐function‐related pathways. Our research found that melatonin can reduce the excessive branching of microglia in the hippocampal dentate gyrus. Morphological changes in microglia are often linked to their function [94]. By quantifying CD68/Iba1‐positive cells, we discovered that melatonin can reduce microglial activation and phagocytosis induced by prenatal stress. We believe that microglia play a critical role in melatonin's mitigation of prenatal stress effects. Further hippocampal sequencing results also indicate that melatonin treatment can reduce the enrichment of microglial genes and the upregulation of immune function‐related pathways.

CXCL10, a member of the CXC chemokine family, functions by binding specifically to the CXCR3 receptor [95]. Under pathological conditions, microglia can be activated and release various cytokines and chemokines, including CXCL10, which participate in disease processes [96, 97]. Recent studies suggest that CXCL10 could be a potential biomarker and therapeutic target for CNS inflammatory responses [98]. Hyperglycemia in neonatal rats can induce CXCL10/CXCR3 signaling, microglial activation, and astrocyte proliferation, altering long‐term synaptic formation and function in the hippocampus [58]. Long‐term exposure of cultured rat hippocampal neurons to CXCL10 can lead to changes in GABA and glutamate receptor protein levels and synaptic network activity [59]. These effects of CXCL10 may contribute to CNS functional changes in chronic neuroinflammatory diseases [60]. Additionally, CXCL10 has been reported to influence the differentiation of hypothalamic embryonic neural precursor cells [61]. In our study, we found that prenatal stress leads to high expression of CXCL10 in the hippocampal dentate gyrus, potentially impairing hippocampal neurodevelopment, and that melatonin can inhibit the expression of CXCL10. In vitro studies show that corticosterone‐treated microglia can inhibit neuronal process growth, while melatonin can partially rescue this growth. To verify the role of CXCL10, we used AM487 to inhibit the CXCR3 receptor in neurons and found that it could rescue the inhibition of neuronal process growth caused by the supernatant from corticosterone‐treated microglia. NF‐κB, a classic inflammatory signaling pathway, is involved in the production of pro‐inflammatory factors and chemokines and can bind to the CXCL10 promoter to regulate its release [99]. We also found that melatonin can inhibit the activation of the NF‐κB signaling pathway in prenatal stress. Therefore, we propose that melatonin may regulate CXCL10 expression through the NF‐κB signaling pathway, thereby affecting neurogenesis and synaptic development in the dentate gyrus region. The PI3K‐AKT pathway is crucial in cellular processes such as cell survival, proliferation, migration, and metabolism [100]. Phosphorylated Akt, a major kinase in this signaling pathway, is involved in producing and synthesizing pro‐inflammatory mediators [101, 102]. In certain inflammatory environments, overactivation of the PI3K‐AKT signaling pathway can exacerbate the inflammatory response, with NF‐κB being a downstream pathway [103, 104, 105]. Our results suggest that by inhibiting the PI3K‐AKT pathway, melatonin regulates the NF‐κB signaling pathway, affecting the polarization state of microglia and the expression of chemokine CXCL10.

## 5 Limitations

This study primarily focuses on the hippocampus, but this focus may limit our understanding of the impact of prenatal stress on other brain regions, such as the amygdala and prefrontal cortex. Therefore, future studies should expand the analysis to include additional brain regions involved in emotional regulation, to more comprehensively elucidate the global effects of melatonin. Additionally, while the in vitro microglial cell culture model is valuable for studying cell‐specific mechanisms, it cannot fully replicate the complex in vivo physiological environment. Future research could consider using coculture systems or brain organoid models, which more accurately simulate the interactions between neurons and glial cells.

## 6 Conclusion

In conclusion, our results indicate that melatonin has significant therapeutic potential for prenatal stress. Melatonin is a safe and effective drug with a favorable safety profile in both daily health care and clinical use. Research on prenatal stress treatment holds substantial public health implications. Our findings expand the potential applications of melatonin and provide guidance for its clinical use.

## Author Contributions

D.W., W.Z., and A.H. conceived and designed the study. D.W. performed the majority of the laboratory work, the analysis of the data, and the writing of the manuscript; J.D. and T.Z. were involved in the collection and assembly of data. N.L., X.Q., F.P., D.W., J.S., and S.Z. contributed to the data analysis and interpretation. C.D. and L.W. provided technical support for the experiment. W.Z. and A.H. were involved in data analysis and interpretation, manuscript writing, financial support, and final approval of the manuscript.

## Ethics Statement

The animal study was reviewed and approved by the National Institutes of Health Guide for the Care and Use of Laboratory Animals and the Institutional Animal Care and Use Committees of Shandong University.

## Conflicts of Interest

The authors declare no conflicts of interest.

## Supporting information


Figure S1.

## Data Availability

The data that support the findings of this study are available from the corresponding author upon reasonable request.
