# Supplementary material for: Melatonin Alleviates Behavioral and Neurodevelopmental Abnormalities in Offspring Caused by Prenatal Stress
Source: CNS Neurosci Ther. 2025 Mar 25;31(3):e70347. doi: 10.1111/cns.70347 (PMC11933876; doi:10.1111/cns.70347)
Supplement: Supplementary file 1 — Figure S1. [file CNS-31-e70347-s001.docx]

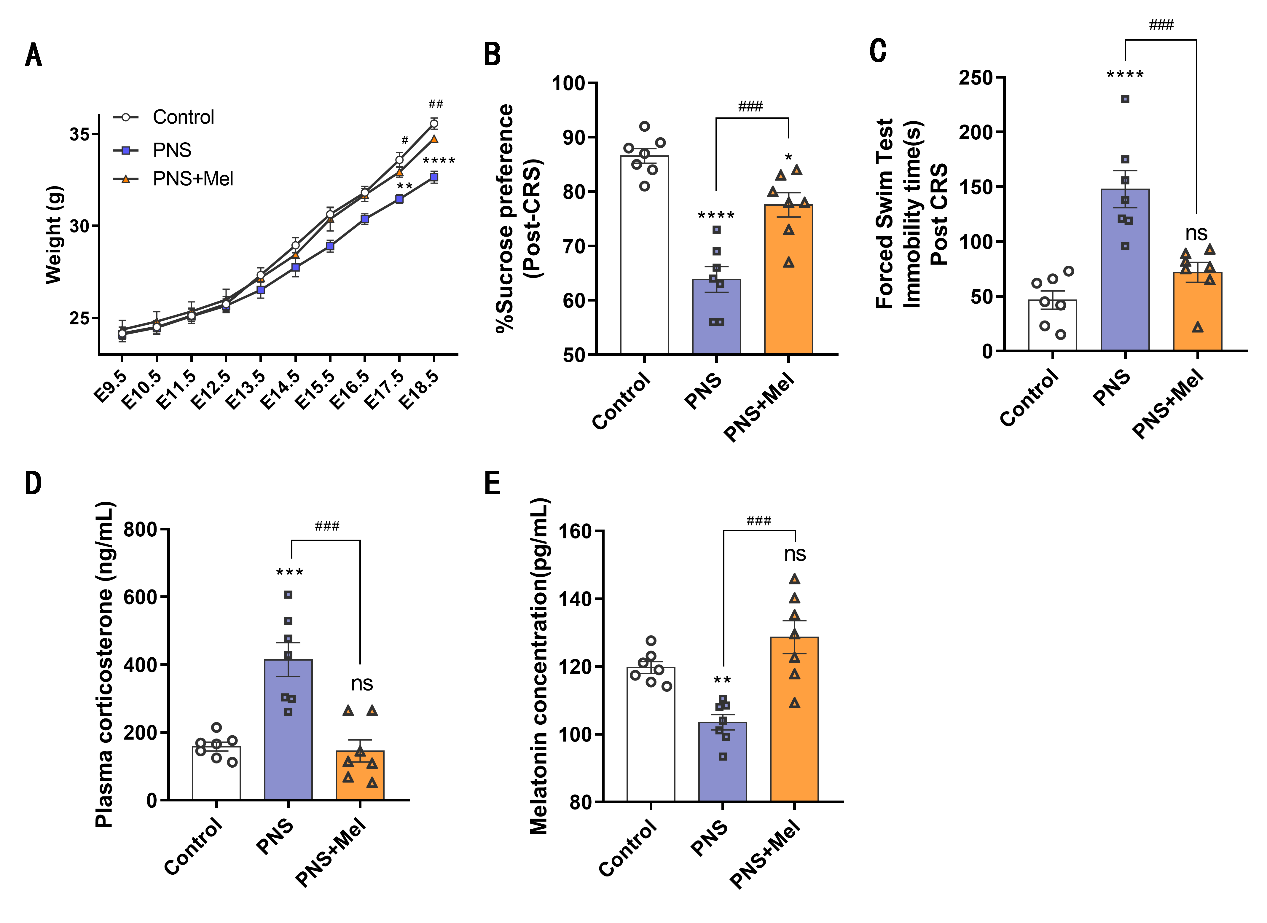


**Supplementary Figure 1. Melatonin mitigated weight loss, depressive mood, elevated plasma corticosterone levels, and decreased melatonin levels in maternal mice induced by prenatal stress.**

A Effects of prenatal stress on body weight gain in pregnant mice. B Effect of different treatment groups on sucrose solution preference (%). C The immobility time of different treatment groups in forced swimming test. D Changes of plasma corticosterone secretion levels in different treatment groups. E Changes of plasma melatonin secretion levels in different treatment groups. n=7 for each group.The results are shown as the mean ± SEM, and analyzed by one-way ANOVA followed by post-hoc Turkey test. **p < 0.01, ***p < 0.001，****p < 0.0001 compared with the Control group. #p < 0.05, ###p < 0.001 compared with the PNS group.


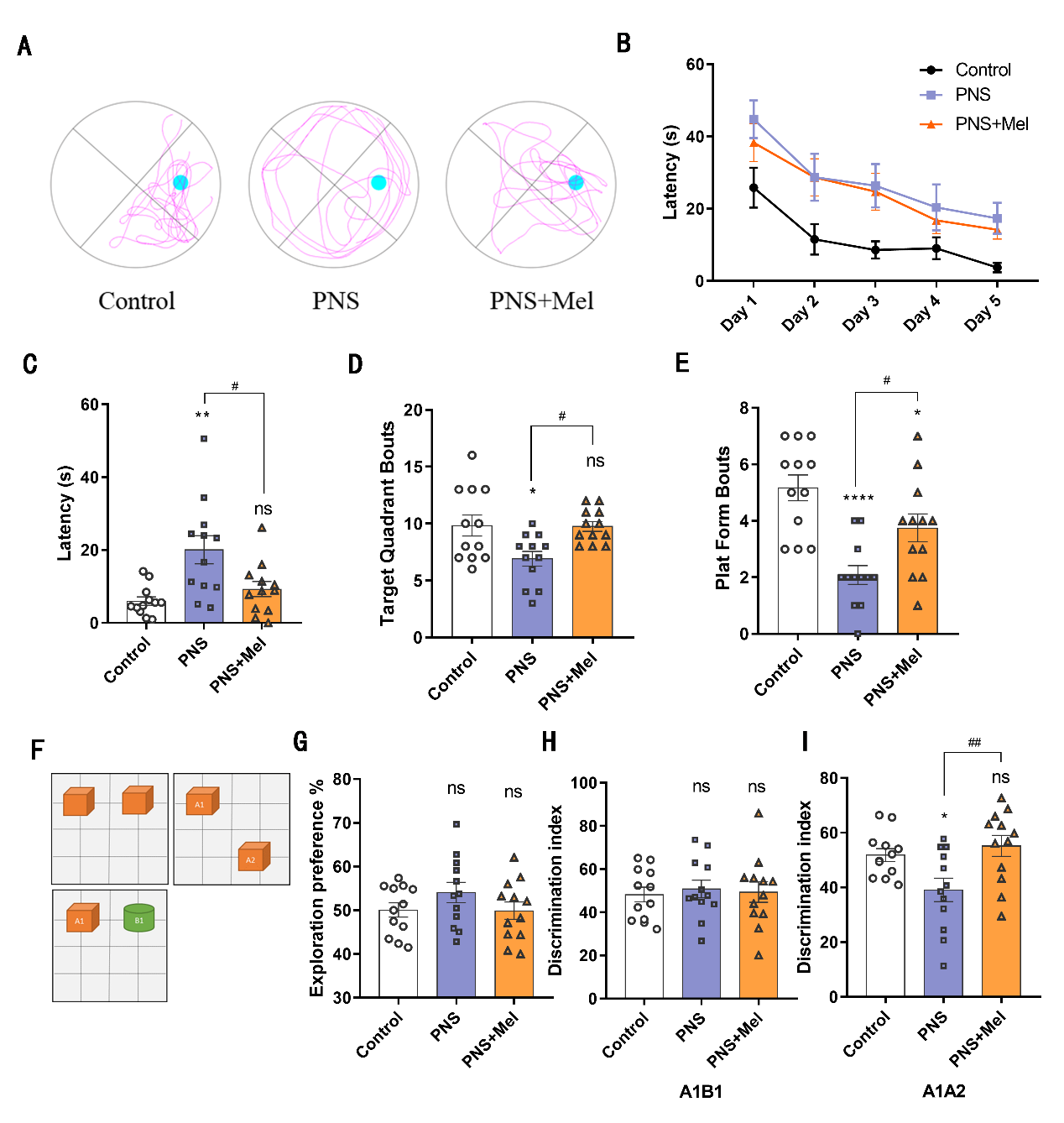


**Supplementary Figure 2. Melatonin ameliorated PNS-induced cognitive impairment in offspring mice.**

A Representative trajectory of mice in MWM. B The latency of different groups of mice entering the target platform during training. C The latency of different groups of mice entering the target platform on the test day. D The number of times that different groups of mice entered the target quadrant on the test day. E The number of times that different groups of mice entered the plat form on the test day. F Schematic representation of NOR. G Exploration preferences of different groups of mice in NOR sample phase. H-I The performance of different groups of mice on object exploration in NOR test phase. n=12 for each group. The results are shown as the mean ± SEM, and analyzed by one-way ANOVA followed by post-hoc Turkey test. *p <0.05，**p < 0.01, ****p < 0.0001 compared with the Control group. #p < 0.05, ##p < 0.01 compared with the PNS group.


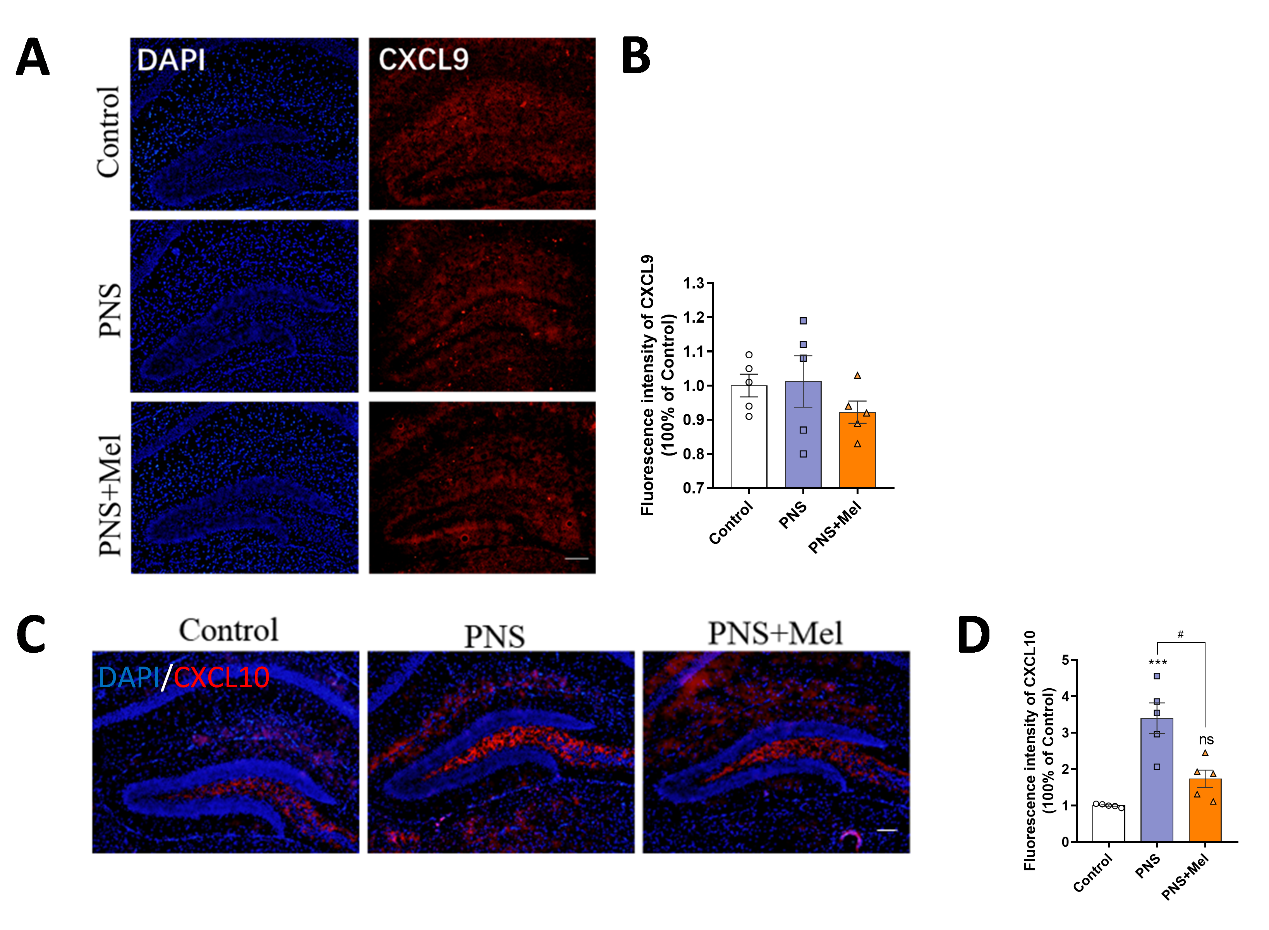


**Supplementary Figure 3. CXCL10 is highly expressed in the dentate gyrus of hippocampus.**

A-B Representative images and quantification analysis immunofluorescence of CXCL9 expression in hippocampal. Scale bars: 75 μm. C-D Representative images and quantification of immunofluorescence analysis of CXCL10 expression in hippocampal. Scale bars: 50 μm. n = 5 for each group. The results are shown as the mean ± SEM, and analyzed by one-way ANOVA followed by post-hoc Turkey test. ***p < 0.001 compared with the Control group. #p < 0.05 compared with the PNS group.


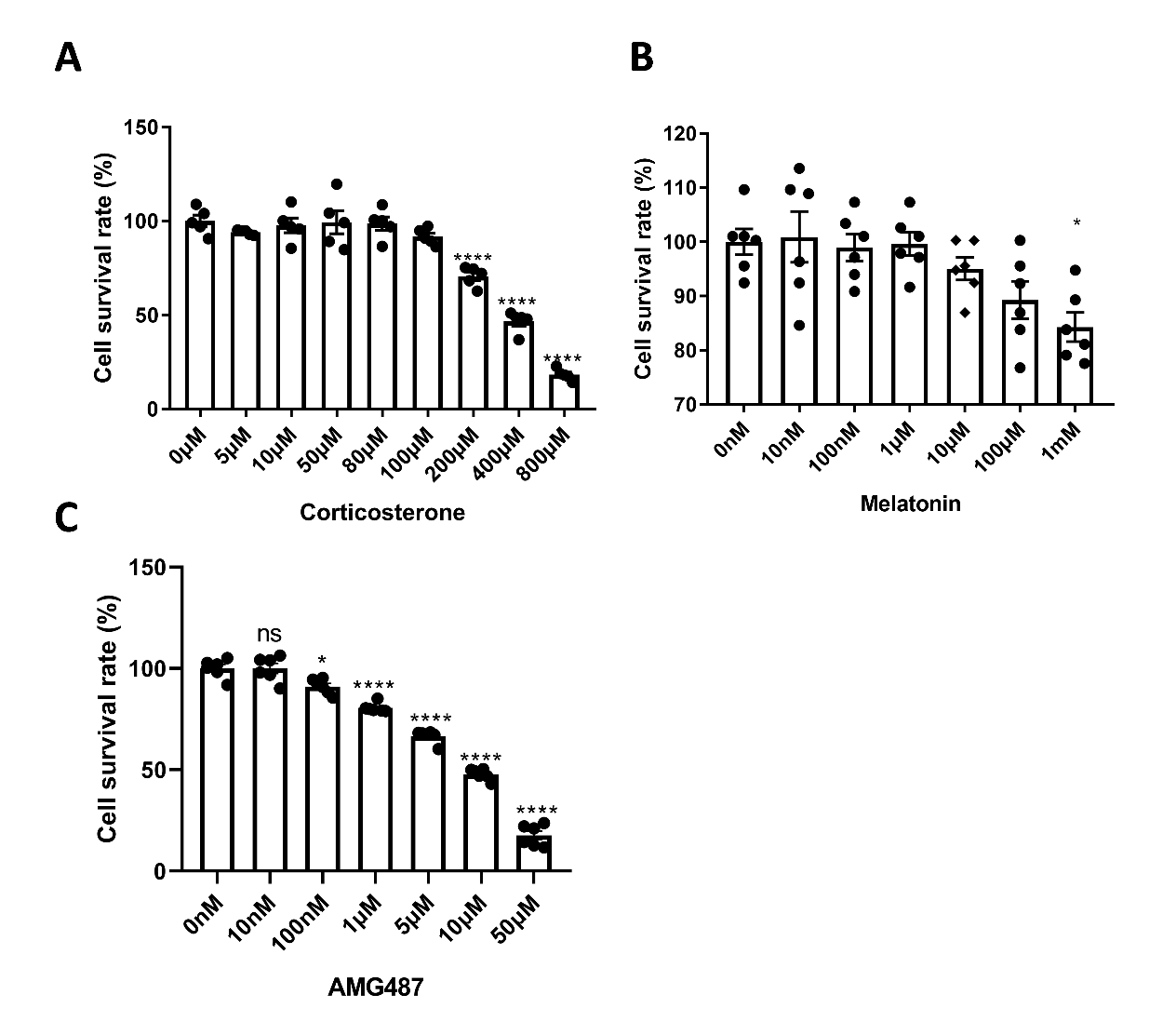


**Supplementary Figure4. Effects of Corticosterone, Melatonin, and AMG487 on Cell Viability Assessed by CCK-8 Assay.**

A. Microglial viability measured using the CCK-8assay after treatment with 5, 10, 50, 80, 100, 200, 400, and 800 μM corticosterone. B. Microglial viability measured using the CCK-8 assay after treatment with 0.01, 0.1, 1, 10, 100, and 1000 μM melatonin. C. N2a cell viability measured using the CCK-8 assay after treatment with 0.01, 0.1, 1, 5, 10, and 50 μM AMG487. The results are shown as the mean ± SEM, and analyzed by one-way ANOVA followed by post-hoc Turkey test. *p <0.05， ****p < 0.0001 compared with the Control group.
